# Stapled versus handsewn methods for colorectal anastomosis surgery: a systematic review of randomized controlled trials

**DOI:** 10.1590/S1516-31802002000500002

**Published:** 2002-09-02

**Authors:** Suzana Angélica da Silva Lustosa, Delcio Matos, Álvaro Nagib Atallah, Aldemar Araujo Castro

**Keywords:** Surgical anastomosis, Systematic review, Meta-analysis, Colorectal surgery, Anastomose cirúrgica, Revisão acadêmica, Metanálise, Cirurgia colorretal

## Abstract

**CONTEXT:**

The interest in the results from comparisons between handsewing and stapling in colorectal anastomoses has been reflected in the progressive increase in the number of clinical trials. These studies, however, do not permit conclusions to be drawn, given the lack of statistical power of the samples analyzed.

**OBJECTIVE:**

To compare stapling and handsewing in colorectal anastomosis, testing the hypothesis that in colorectal anastomosis the technique of stapling is superior to that of handsewing.

**DESIGN:**

A systematic review of randomized controlled trials.

**SURVEY STRATEGY:**

Systematic revision of the literature and meta-analysis were used, without restrictions on language, dates or other considerations. The sources of information used were Embase, Lilacs, Medline, Cochrane Controlled Clinical Trials Database, and letters to authors and industrial producers of staples and thread.

**SELECTION CRITERIA:**

Studies were included in accordance with randomization criteria. The external validity of the studies was investigated via the characteristics of the participants, the interventions and the variables analyzed. An independent selection of clinical studies focusing on analysis of adult patients attended to on an elective basis was made by two reviewers.

**DATA COLLECTION AND ANALYSIS:**

The methodological quality of the studies was assessed by the same reviewers. In addition to the randomization criteria, the masking, treatment intention, losses and exclusions were also analyzed. The meta-analysis was performed using risk difference and weighted average difference, with their respective 95% confidence intervals. The variables studied were mortality, clinical and radiological anastomotic dehiscence, anastomotic stricture, hemorrhage, reoperation, wound infection, time taken to perform the anastomosis and hospital stay.

**RESULTS:**

Nine clinical trials were selected. After verifying that it was possible to perform one of the two techniques being compared, 1,233 patients were included, of whom 622 underwent stapling and 611 the handsewing technique. No statistical difference was found between the variables, except for stenosis, which was more frequent in stapling (p < 0.05), and the time taken to perform the anastomosis, which was greater in handsewing (p < 0.05).

**CONCLUSION:**

The evidence found was insufficient to demonstrate superiority of the stapling method over handsewing, independent of the level of colorectal anastomosis.

## RESUMO

**CONTEXTO:**

O aumento do número de ensaios clínicos tem demonstrado o alto interesse nos resultados de comparações entre sutura manual e grampeamento nas anastomoses colorretais. Esses estudos, no entanto, não permitem conclusões em virtude da falta de poder estatístico das amostras analisadas.

**OBJETIVO:**

Comparar anastomoses colorretais realizadas com sutura manual e com grampeamento, testando a hipótese de que a técnica que utiliza o grampeador é mais vantajosa em relação aquela realizada com fios de sutura.

**TIPO DE ESTUDO:**

Revisão sistemática de ensaios clínicos randomizados e controlados.

**ESTRATÉGIA DA PESQUISA:**

Uma revisão sistemática da literatura foi realizada, sem restrições de língua, datas ou outras considerações. As fontes de informação utilizadas foram Embase, Lilacs, Medline, Base de Dados de Ensaios Clínicos Controlados da Colaboração Cochrane e cartas para autores e produtores de grampos e fios de sutura.

**CRITÉRIOS DE SELEÇÃO:**

Os estudos foram incluídos na amostra de acordo com os critérios de randomização. A validade externa das pesquisas foi investigada pelas características dos participantes, pelas intervenções e pelas variáveis analisadas. Dois revisores realizaram a seleção dos estudos clínicos, os quais enfocaram análises de pacientes adultos atendidos eletivamente.

**COLETA DE DADOS E ANÁLISE:**

A qualidade metodológica dos estudos foi investigada pelos mesmos revisores. Além disso, os critérios de randomização, o mascaramento, a intenção de tratamento, perdas e exclusões foram também analisadas. A metanálise foi realizada utilizando-se diferença de risco e diferença de média ponderal, com os respectivos intervalos de confiança de 95%. As variáveis estudadas foram mortalidade, deiscência, estenose, hemorragia, reoperação, infecção da parede abdominal, tempo de realização da anastomose e tempo de internação.

**RESULTADOS:**

Nove ensaios clínicos foram selecionados. Após a constatação de que era possível a utilização de uma das duas técnicas que estavam sendo comparadas, 1.233 pacientes foram incluídos, dos quais 622 foram submetidos à técnica do grampeamento e 611 à técnica de sutura manual com fios. As diferenças encontradas entre as variáveis não foram significantes, exceto para a estenose, que foi mais freqüente na técnica do grampeamento (p < 0,05) e tempo de realização da anastomose que foi maior para a técnica de sutura manual (p < 0,05).

**CONCLUSÃO:**

As evidências encontradas foram insuficientes para demonstrar superioridade da técnica de grampeamento sobre a técnica de sutura manual nas anastomoses colorretais, independentemente do nível da anastomose.

## INTRODUCTION

The interest in the results from comparisons between handsewing and stapling has been progressively growing. However, the majority of the results come from studies of inadequate methodological quality. Looking only at the conclusions from randomized studies, it can be seen that the results are not uniform.^1–9^ In considering whether the level of the anastomosis is supra or infra-peritoneal, the controversy is further widened.^1,10–12^

A systematic review and meta-analysis of 17 studies comparing handsewing and stapling in ileocolonic, colocolonic and colorectal anastomoses was done by MacRae & McLeod^13^ in 1998. They concluded that although intraoperative technical problems were more common in those that were stapled, no evidence of differences between the two groups was found in the other variables, and they considered the two techniques to be effective. Systematic reviews that exclusively included comparisons of colorectal anastomosis have not been found.

The accomplishment of a systematic review is the best manner for describing the state of our knowledge, and hence becomes the best way of obtaining quality scientific evidence. Consequently, this systematic review and meta-analysis was proposed, so as to analyze the results from clinical trials that compared only colorectal anastomoses done using stapling or handsewing.

## METHODS

The method of systematic review with meta-analysis of randomized clinical trials was used, making comparisons between stapling and handsewing in elective colorectal anastomoses in adult patients. The mechanical suturing was always performed using a circular stapler and there was no restriction as to the anastomosis technique or material used in manual suturing. The studies were located via electronic databases: registers of randomized clinical trials of the colorectal cancer group of the *Cochrane Collaboration, Medline*, *Embase, Lilacs* and contact with authors and industrial producers of staples and threads. An evaluation of the methodology of the clinical trials was performed, with the objective of accessing the applicability of the findings, validity of the individual studies and the characteristics of the research model.

The inclusion or non-inclusion of a study depended on the evaluation of the randomization. The most important criterion for the classification was the secrecy of the allocation, which should be maintained until the time of the intervention.^14^ The data were collected using software from the *Cochrane Collaboration*: *Review Manager, Version 4.0 for Windows, Oxford (UK).*

The variables analyzed were: mortality, anastomotic dehiscence, stenosis, hemorrhage, reoperation, infection of the operative wound, time taken to perform the anastomosis and length of hospitalization. Anastomotic dehiscence was regarded as the most important variable to be taken into consideration.

When appropriate, the studies were placed in subgroups for performing the meta-analysis according to the anastomotic level (supra or infra-peritoneal). For dichotomous variables, the meta-analysis was effected in accordance with the method of risk difference for a random model and number needed for treatment. For the continuous variables, the weighted average difference was used. The respective confidence interval for all these was 95%.^15^ The date of the last search for clinical trials for this systematic review was December 2001.

## RESULTS

Nine randomized controlled clinical trials comparing stapling with handsewing anastomosis were found in the literature search. Thus, 1233 patients were studied after verifying that the use of both techniques would have been possible: 622 underwent the stapling and 611 the handsewing anastomotic technique. The clinical trials included in the systematic review, the sample sizes and the levels of the colorectal anastomoses are shown in Table 1.

**Table 1:** Clinical trials included in the systematic review and meta-analysis

Authors	Sample	Stapled	Handsewn	Anastomosis Levels
Beart et al., 1981^1^	70	35	35	supra-peritoneal
Elhadad et al., 1990^6^	272	139	133	not specified
Fingerhut et al., 1994^11^	113	54	59	supra-peritoneal
Fingerhut et al., 1995^12^	159	85	74	supra-peritoneal
Gonzalez et al., 1989^5^	113	55	58	not specified
Kracht et al., 1991^8^	268	137	131	not specified
McGinn et al., 1985^3^	118	58	60	infra-peritoneal
Sarker et al., 1994^9^	60	30	30	supra-peritoneal
Thiede et al., 1984^2^	60	29	31	not specified

The mortality results were based on 7 studies that included 901 patients, in which 2.4% (11/453) of the handsewn group and 3.6% (16/448) of the stapled group died. No significant statistical difference was shown, with a 95% confidence interval of −2.8 to 1.6% and a risk difference of −0.6%.

Overall anastomotic dehiscence that included clinical and radiological anastomotic breakdown was analyzed in 9 studies that included 1,233 patients: 81 out of 622 (13.0%) and 82 out of 611 (13.4%) patients in the handsewn and stapled groups respectively showed this complication. No significant statistical difference was found, with a risk difference of 0.2% and a 95% confidence interval of −5.0 to 5.3%.

Clinical anastomotic dehiscence results were based on 9 studies that included 1,233 patients, among whom 39 (626) were in the handsewn group (6.33%) and 44 (617) in the stapled group (7.1%); no significant statistical difference was found, with a risk difference of −1.4% and a 95% confidence interval of −5.2 to 2.3%.

Radiological anastomotic dehiscence was analyzed in 6 studies that included 825 patients: 33 out of 421 (7.8%) and 30 out of 414 (7.2%) in the handsewn and stapled groups respectively showed this finding. No significant statistical difference was found, with a risk difference of 1.2% and a 95% confidence interval of −4.8 to 7.3%.

Anastomotic stricture results were based on 7 studies that included 1042 patients, of whom 8% (40/500) were in the handsewn group and 2% (10/496) in the stapled group. A significant statistical difference was found (p = 0.00001), favoring the handsewn technique, with a risk difference of 4.6% and a 95% confidence interval of 12 to 31%.

Hemorrhage from the anastomotic site was analyzed in 4 studies that included 662 patients: 5.4% (18/336) and 3.1% (10/326) in the handsewn and stapled groups respectively showed this complication. No significant statistical difference was found, with a risk difference of 2.7% and a 95% confidence interval of −0.1 to 5.5%.

Reoperation of the patients after anastomotic complications was analyzed in 3 studies that included 544 patients: 7.6% (21/278) and 4.1% (11/266) of the handsewn and stapled groups respectively. No significant statistical difference was found, with a risk difference of 3.9% and a 95% confidence interval of 0.3 to 7.4%.

Wound infection was analyzed in 6 studies that included 567 patients: 5.9% (17/286) and 4.3% (12/282) of the handsewn and stapled groups respectively. No significant statistical difference was found, with a risk difference of 1.0% and a 95% confidence interval of −2.2 to 4.3%.

Anastomosis duration, or the time taken to perform the anastomosis, was analyzed as a continuous outcome in just 1 study that included 159 patients. The weighted mean difference value was −7.6 minutes, with a 95% confidence interval of −12.9 to −2.2. This result showed a significant statistical difference (p = 0.005), favoring the stapling technique.

The length of hospital stay was also analyzed as a continuous variable, in 1 study that included 159 patients. The average value found was 2.0 days, and no significant statistical difference was found, with a 95% confidence interval of −3.2 to 7.2.

A summary of the meta-analysis results for each variable is presented in Table 2, with the number of studies included, the number of participants, and the results from the heterogeneity and overall effect tests.

**Table 2:** Summary of the meta-analysis results

Clinical outcome	Number of studies	Number of participants	Statistical Methods	Effect sixe	Test for heterogeneity	Test for overall effect
Mortality	7	901	Peto OR (95% Cl)	0.69 (0.32 to 1.49)	Chi-squared 7.10 df = 6 P = 0.31	Z = −0.94 P = 0.3
Overall dehiscence	9	1,233	Peto OR (95% Cl)	0.99 (0.71 to 1.40)	Chi-squared 15.84 df = 8 P = 0.045	Z = −0.03 P = 1
Clinical dehiscence	10	1,233	Peto OR (95% Cl)	0.80 (0.51 to 1.24)	Chi-squared 973 df = 7 P = 0.2	Z = −0.99 P = 0.3
Radiological dehiscence	6	835	Peto OR (95% Cl)	1.10 (0.66 to 1.85)	Chi-squared 13.89 df = 5 P = 0.016	Z = 0.37 P = 0.7
Stricture	7	996	Peto OR (95% Cl)	3.59 (2.02 to 6.35)	Chi-squared 4.80 df = 5 P = 0.44	Z = 4.38 P = 0.00001
Hemorrhage	4	662	Peto OR (95% Cl)	1.78 (0.84 to 3.81)	Chi-squared 4.65 df = 3 P = 0.2	Z = 1.49 P = 0.14
Reoperation	3	544	Peto OR (95% Cl)	1.94 (0.95 to 3.98)	Chi-squared 1.73 df = 2 P = 0.42	Z = 1.81 P = 0.07
Wound infection	6	568	Peto OR (95% Cl)	1.43 (0.67 to 3.04)	Chi-squared 4.12 df = 5 P = 0.53	Z = 0.93 P = 0.4
Anastomotic duration	1	159	WMD [Fixed] (95% Cl)	-7.60 (-12.92 to-2.28)	Chi-squared 0.0 df = 0	Z = 2.80 P = 0.005
Hospital stay	1	159	WMD [Fixed] (95% Cl)	2.00 (-3.27 to 7.27)	Chi-squared 0.0 df = 0	Z = 0.74 P = 0.5

*CI = confidence interval; OR = odds ratio; WMD = weight mean difference; df = degrees of freedom; p = p-value.*

**Figure 1:**
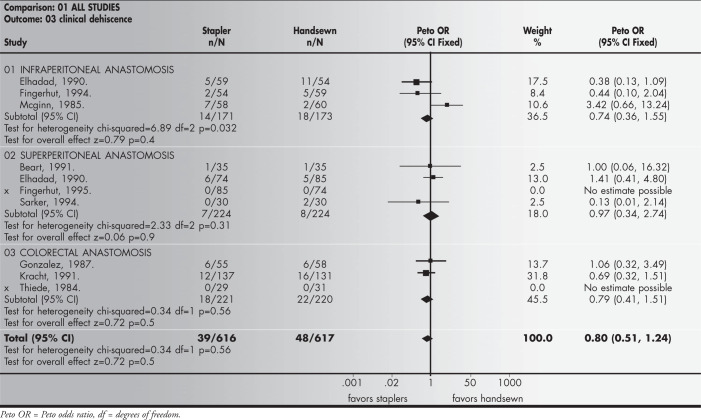
Anastomotic clinical dehiscence — Meta-analysis.

The results from the meta-analysis of the anastomotic clinical dehiscence, regarded as the main variable of this study, are shown in Figure 1.

## DISCUSSION

The basic premise of this systematic review was that by grouping studies without statistical power but with methodological quality, a sample large enough to show up any possible significant differences could be obtained. This was not observed, given that the majority (7/9) of the variables analyzed were not significantly different. Perhaps this fact can be explained by insufficient sample sizes for demonstrating the magnitude of the difference formulated, which highlights the necessity for periodic updating of this review.

The data relating to mortality show that clinical dehiscence was responsible for four deaths in the group sutured by stapling, and for two deaths in the handsewn group. The anastomotic dehiscence, evaluated clinically and radiologically, did not show a significantly different incidence between the techniques of stapling and handsewing. Infra-peritoneal colorectal anastomoses are considered to be higher in risk, accepting that more distal anastomoses are frequent hosts for this complication.^17,18^

The classification criteria for colorectal anastomoses in relation to this were not uniform in the literature surveyed in this review. Thus, some authors refer to endoscopic measurements for classifying anastomoses,^1,3,11,12^ while others classify anastomoses as high or low without referring to the criteria adopted.^8^

In several non-randomized studies, a greater incidence of stenosis is attributed to the technique of stapling.^21–24^ In the present systematic review it was observed that the length of follow-up for the stenosis parameter varied a lot between the different studies, which made comparison excessively difficult and may have made the overall result imprecise.

In this systematic review, it was also noted that stenosis occurred to a significant extent in patients submitted to colorectal anastomosis using stapling, especially in infra-perito-neal anastomoses. This scientific evidence may be considered relevant in this survey. However, the majority of the studies (7/9) considered this complication to be irrelevant from a clinical point of view, given the favorable evolution with conservative treatment in the great majority of the cases. A statistically significant difference in the results, favoring the handsewing technique, was thus expressed, albeit without there having been any relevant clinical difference between the two techniques. One explanation for the mismatch between the statistical and clinical differences is the possible lack of methodological adequacy in the studies.

The time taken to perform the anastomosis was significantly shorter in colorectal anastomoses done with the stapler than when handsewing was done. A limitation to the analysis of this variable was that only one study^12^ provided data (average and standard deviation) that could be introduced into the program for later statistical analysis. The time taken to perform the anastomosis has a relative value when analyzed in isolation, i.e. when not associated with the total length of the operative procedures or hospitalization of the patient. The other variables analyzed did not demonstrate any advantage of one technique over the other.

Comparing anastomoses performed by handsewing with others that are stapled, the former depend intrinsically on the ability of the surgeon, since their execution requires appropriate introduction of the needle via the layers of the intestinal wall and uniform spacing between passages of the suturing thread, among other maneuvers. In stapling, the anastomosis device performs these procedures in a uniform and automatic manner.

Various procedures may alter the security of a colorectal anastomosis. Protection colostomy,^19^ epiploplasty, complementary suturing and the performance or otherwise of an integrity test on the anastomosis^20^ are procedures frequently used by surgeons, but not in a uniform manner. In this review, the great majority of authors (8/9) used such procedures, which could interfere in the final results. It can be considered that when these manipulations are employed, the patients should be analyzed in a stratified manner.

It is possible that the results have been in some way influenced by aspects of a learning curve related to any differences in experience between surgeons participating in these studies. In addition to this learning curve, another factor related to the results from colorectal anastomoses is the adequate functioning of the instrument used. In this systematic review, some authors^1,3,11^ analyzed this aspect together with the experience of the surgeon. It is accepted that these two parameters, the failure of instruments and the experience of surgeons, should be analyzed independently.^16^

The question of cost, which was not analyzed in this survey, is related to the length of the operative procedure, length of hospitalization, price of thread and value of devices used, among other factors. This represents a variable of great importance, deserving special attention in studies with that specific objective. It is also considered that a more detailed study of costs in this review would become necessary in the event of evidence that the stapling technique was more advantageous. When only the cost of the material used in the anastomosis is taken into consideration, the stapler is more expensive. The cost of an operative procedure, however, must be analyzed within a wider context involving not only the monetary value of the materials but also the value resulting from the ease of execution, total time consumed, cost of complications related to the method employed, among other factors.

With regard to implications of a practical nature, the evidence encountered is insufficient to demonstrate the superiority of the stapling method over handsewing. The decision on which technique to use must remain at the discretion of an appropriate judgement by the surgeon, i.e. based on his personal experience, circumstantial facts and resources available. From the point of view of clinical research, it will be necessary for clinical trials developed in relation to this question to involve criteria that respect the representativeness of the sample, rigorous definition and standardization of the variables, appropriate statistical treatment of the data and stratification of risky anastomoses.

A more detailed review is published and updated in the Cochrane database of systematic reviews.^25^

## CONCLUSION

The results of the present systematic review of the literature and meta-analysis permit the conclusion that the evidence encountered is insufficient to demonstrate superiority of the stapling method in relation to handsewing in colorectal anastomosis, independent of the level of the colorectal anastomosis.
